# SciPipe: A workflow library for agile development of complex and dynamic bioinformatics pipelines

**DOI:** 10.1093/gigascience/giz044

**Published:** 2019-04-26

**Authors:** Samuel Lampa, Martin Dahlö, Jonathan Alvarsson, Ola Spjuth

**Affiliations:** 1 Department of Pharmaceutical Biosciences and Science for Life Laboratory, Uppsala University, Box 591, 751 24, Uppsala, Sweden; 2 Department of Biochemistry and Biophysics, National Bioinformatics Infrastructure Sweden, Science for Life Laboratory, Stockholm University, Svante Arrhenius väg 16C, 106 91, Solna, Sweden

**Keywords:** scientific workflow management systems, pipelines, reproducibility, machine learning, flow-based programming, Go, Golang

## Abstract

**Background:**

The complex nature of biological data has driven the development of specialized software tools. Scientific workflow management systems simplify the assembly of such tools into pipelines, assist with job automation, and aid reproducibility of analyses. Many contemporary workflow tools are specialized or not designed for highly complex workflows, such as with nested loops, dynamic scheduling, and parametrization, which is common in, e.g., machine learning.

**Findings:**

SciPipe is a workflow programming library implemented in the programming language Go, for managing complex and dynamic pipelines in bioinformatics, cheminformatics, and other fields. SciPipe helps in particular with workflow constructs common in machine learning, such as extensive branching, parameter sweeps, and dynamic scheduling and parametrization of downstream tasks. SciPipe builds on flow-based programming principles to support agile development of workflows based on a library of self-contained, reusable components. It supports running subsets of workflows for improved iterative development and provides a data-centric audit logging feature that saves a full audit trace for every output file of a workflow, which can be converted to other formats such as HTML, TeX, and PDF on demand. The utility of SciPipe is demonstrated with a machine learning pipeline, a genomics, and a transcriptomics pipeline.

**Conclusions:**

SciPipe provides a solution for agile development of complex and dynamic pipelines, especially in machine learning, through a flexible application programming interface suitable for scientists used to programming or scripting.

## Findings

Driven by the highly complex and heterogeneous nature of biological data [1,2], computational biology is characterized by an extensive ecosystem of command-line tools, each specialized on one or a few of the many aspects of biological data. Because of their specialized nature these tools generally need to be assembled into sequences of processing steps, often called “pipelines,” to produce meaningful results from raw data. As a result of the increasingly large sizes of biological data sets [3,4], such pipelines often require integration with high-performance computing (HPC) infrastructures or cloud computing resources to complete in an acceptable time. This has created a need for tools to coordinate the execution of such pipelines in an efficient, robust. and reproducible manner. This coordination can in principle be done with simple scripts in languages such as Bash, Python, or Perl, but plain scripts can quickly become fragile. When the number of tasks becomes sufficiently large and the execution times long, the risk for failures during the execution of such scripts increases almost linearly with time, and simple scripts are not a good strategy for when large jobs need to be restarted from a failure. This is because they lack the ability to distinguish between finished and half-finished files. They also do not provide means to detect whether intermediate output files are already created and can be reused to avoid wasting time on redoing already finished calculations. These limitations with simple scripts call for a strategy with a higher level of automation and more careful management of data and state. This need is addressed by a class of software commonly referred to as “scientific workflow management systems” or simply “workflow tools.” Through a more automated way of handling the execution, workflow tools can improve the robustness, reproducibility, and understandability of computational analyses. In concrete terms, workflow tools provide means for handling atomic writes (making sure finished and half-finished files can be separated after a crashed or stopped workflow), caching of intermediate results, distribution of tasks to the available computing resources, and automatically keeping or generating records of exactly what was run, to make analyses reproducible.

It is widely agreed that workflow tools generally make it easier to develop automated, reproducible, and fault-tolerant pipelines, although many challenges and potential areas for improvement persist with existing tools [5]. This has made scientific workflow systems a highly active area of research. Numerous workflow tools have been developed, and many new ones are continually being developed.

The workflow tools developed differ widely in terms of how workflows are defined and what features are included out-of-the-box. This probably reflects the fact that different types of workflow tools can be suited for different categories of users and use cases. Graphical tools such as Galaxy [6,7] and Yabi [8] provide easy-to-use environments especially well-suited for scientists without scripting experience. Text-based tools such as Snakemake [9], Nextflow [10], BPipe [11], Cuneiform [12], and Pachyderm [13], on the other hand, are implemented as domain-specific languages (DSLs), which can often provide a higher level of flexibility, at the expense of the ease of use of a graphical user interface. They can thus be well-suited for “power users” with experience in scripting or programming.

Even more power and flexibility can be gained from workflow tools implemented as programming libraries, which provide their functionality through a programming application programming interface (API) accessed from an existing programming language such as Python, Perl, or Bash. By implementing the API in an existing language, users get access to the full power of the implementation language as well as the existing tooling around the language. One example of a workflow system implemented in this way is Luigi [14]. Another interesting workflow system implemented as a programming library and that shares many features with SciPipe is Nipype [15].

As reported by Spjuth et al. [5], although many users find important benefits in using workflow tools, many also experience limitations and challenges with existing tools, especially regarding the ability to express complex workflow constructs such as branching and iteration, as well as limitations in terms of audit logging and reproducibility. Below we briefly review a few existing popular systems and highlight areas where we found that the development of a new approach and tool was desirable, for use cases that include very complex workflow constructs.

First, graphical tools such as Galaxy and Yabi, although easy to use even without programming experience, are often perceived to be limited in their flexibility owing to the need to install and run a web server to use them, which is not always permitted or practical on HPC systems. Text-based tools implemented as DSLs, such as Snakemake, Nextflow, BPipe, Pachyderm, and Cuneiform, do not have this limitation but have other characteristics that might be problematic for for complex workflows.

For example, Snakemake is dependent on file-naming strategies for defining dependencies, which can in some situations be limiting, and also uses a “pull-based” scheduling strategy (the workflow is invoked by asking for a specific output file, whereafter all tasks required for reproducing the file will be executed). While this makes it easy to reproduce specific files, it can make the system hard to use for workflows involving complex constructs such as nested parameter sweeps and cross-validation fold generation, where the final file names are difficult if not impossible to foresee. Snakemake also performs scheduling and execution of the workflow graph in separate stages, meaning that it does not support dynamic scheduling.

Dynamic scheduling, which basically means on-line scheduling during the workflow execution [16], is useful both where the number of tasks is unknown before the workflow is executed and where a task needs to be scheduled with a parameter value obtained during the workflow execution. An example of the former is reading row by row from a database, splitting a file of unknown size into chunks, or processing a continuous stream of data from an external process such as an automated laboratory instrument. An example of the latter is training a machine learning model with hyper parameters obtained from a parameter optimization step prior to the final training step.

BPipe constitutes a sort of middle ground in terms of dynamic scheduling. It supports dynamic decisions of what to run by allowing execution-time logic inside pipeline stages, as long as the structure of the workflow does not need to change. Dynamic change of the workflow structure can be important in workflows for machine learning, however, e.g., if parametrizing the number of folds in a cross-validation based on a value calculated during the workflow run, such as data set size.

Nextflow has push-based scheduling and supports dynamic scheduling via the dataflow paradigm and does not suffer from this limitation. It does not, however, support creating a library of reusable workflow components. This is because of its use of dataflow variables shared across component definitions, which requires processes and the workflow dependency graph to be defined together.

Pachyderm is a container-based workflow system that uses a JavaScript Object Notation (JSON) and YAML-based DSL to define pipelines. It has a set of innovative features including a version-controlled data management component with Git-like semantics and support for triggering of pipelines based on data updates, among others. These in combination can provide some aspects of dynamic scheduling. On the other hand, the more static nature of the JSON/YAML-based DSL might not be optimal for really complex setups such as creating loops or branches based on parameter values obtained during the execution of the workflow. The requirement of Pachyderm to be run on a Kubernetes [17] cluster can also make it less suitable for some academic environments where the ability to run pipelines also on traditional HPC clusters is required. On the other hand, because of the easy incorporation of existing tools, it is possible to provide such more complex behavior by including a more dynamic workflow tool as a workflow step inside Pachyderm instead. We thus primarily see Pachyderm as a complement to other lightweight workflow systems, rather than necessarily an exclusive alternative.

The usefulness of such an approach where an overarching framework provides primarily an orchestration role while calling out to other systems for the actual workflows is demonstrated by the Arteria project [18]. Arteria builds on the event-based StackStorm framework to allow triggering of external workflows based on any type of event, providing a flexible automation framework for sequencing core facilities.

Another group of workflow tools are those designed around the Common Workflow Language (CWL) [19] as their primary authoring interface. These include Toil [20] and Rabix [21], as well as the CWL reference implementation. A detailed review of each of these tools is outside the scope for this article. We note, however, that while CWL provides important benefits in terms of workflow portability, it can at the same time be too limited for very complex and dynamic workflow constructs because of its declarative YAML-based nature, just as for Pachyderm.

There is also a class of workflow systems building on Big Data technologies such as the Hadoop ecosystem, where ADAM [22] is one prominent example, using Spark [23] as a foundation for its pipeline component. Through a set of specialized formats, APIs, and workflow step implementations for genomics data, ADAM manages to provide impressive scalability of genomics analyses across multiple compute nodes. By relying on Spark, which has a programming model in which data operations are expressed directly, ADAM is quite a different beast than the other workflow systems reviewed here, however. In these other more traditional workflow tools, components are generally instead handled in a black-box fashion and most often are implemented outside the workflow layer itself. Just as with Pachyderm, the requirement for additional technology layers for distributed computing, such as the Hadoop distributed file system (HDFS) [24] and the Spark execution system, means that ADAM might not always be a feasible solution for HPC clusters with tight restrictions on system privileges or for local laptops with limited resources.

Returning to traditional workflow systems, Cuneiform takes a different approach than most workflow tools by wrapping shell commands in functions in a flexible functional language (described by Brandt et al. [25]), which allows leveraging common benefits in functional programming languages, such as side effect free functions, to define workflows. It also leverages the distributed programming capabilities of the Erlang virtual machine to provide automatic distribution of workloads. It is still a new DSL, however, which means that tooling and editor support might not be as extensive as for an established programming language.

Luigi is a workflow library developed by Spotify, which provides a high degree of flexibility owing to its implementation as a programming library in Python. For example, the programming API provides full control over file name generation. Luigi also provides integration with many Big Data systems such as Hadoop and Spark, and cloud-centric storage systems such as HDFS and S3.

SciLuigi [26] is a wrapper library for Luigi, previously developed by us, which introduces a number of benefits for scientific workflows by leveraging selected principles from flow-based programming (FBP) (named ports and separate network definition) to achieve an API that makes iteratively changing the workflow connectivity easier than in vanilla Luigi.

While Luigi and SciLuigi have been shown to be a helpful solution for complex workflows in drug discovery, they also have a number of limitations for highly complex and dynamic workflows. First, because Python is an untyped, interpreted language, certain software bugs are discovered only far into a workflow run, rather than while the program is being compiled. Second, the fact that Luigi creates separate processes for each worker that communicate with the central Luigi scheduler via HTTP requests over the network can lead to robustness problems when a certain number of workers is exceeded (∼64 in our experience), leading to HTTP connection timeouts.

Finally Nipype, which is also a programming library implemented in Python and which shares a number of features with SciPipe such as flexible file name generation, separate named inputs and outputs, and a rather FBP-like dependency definition, is expected to have some of the same limitations as Luigi because of the lack of static typing and worse performance of the Python programming language compared to Go.

The aforementioned limitations for complex workflows in existing tools are the background and motivation for developing the SciPipe library.

### The SciPipe workflow library

SciPipe (SciPipe, RRID:SCR_017086) is a workflow library based on FBP principles, implemented as a library in the Go programming language. The library is freely available as open source on GitHub [27]. All releases available on GitHub are also archived on Zenodo [28]. Similarly to Nextflow, SciPipe leverages the dataflow paradigm to achieve dynamic scheduling of tasks based on input data, allowing many workflow constructs not easily coded in many other tools.

Combined with design principles from FBP such as separate network definition and named ports bound to processes, this has resulted in a productive and discoverable API that enables agile authoring of complex and dynamic workflows. The fact that the workflow network is defined separately from processes enables workflows to be built on the basis of a library of reusable components, although the creation of ad hoc shell-command−based components is also supported.

SciPipe provides a provenance tracking feature that creates 1 audit log per output file rather than only 1 for the whole workflow run. This means that it is always easy to verify exactly how each output of a workflow was created.

SciPipe also provides a few features that are not very common among existing tools or that do not commonly occur together in 1 system. These include support for streaming via Unix named pipes, the ability to run push-based workflows up to a specific stage of the workflow, and flexible support for file naming of intermediate data files generated by workflows.

By implementing SciPipe as a library in an existing language, the language’s ecosystem of tooling, editor support, and third-party libraries can be directly used to avoid “reinventing the wheel” in these areas. By leveraging the built-in concurrency features of Go, such as go-routines and channels, the developed code base has been kept small compared with similar tools and also does not require external dependencies for basic use (some external tools are used for optional features such as PDF generation and graph plotting). This means that the code base should be possible to maintain for a single developer or small team and that the code base is practical to include in workflow developers’ own source code repositories, in order to future-proof the functionality of workflows.

Below, we first briefly describe how SciPipe workflows are created. We then describe in some detail the features of SciPipe that are the most novel or improve most upon existing tools, followed by a few more commonplace technical considerations. We finally demonstrate the usefulness of SciPipe by applying it to a set of case study workflows in machine learning for drug discovery and next-generation sequencing genomics and transcriptomics.

### Writing workflows with SciPipe

SciPipe workflows are written as Go programs, in files ending with the .go extension. As such, they require the Go tool chain to be installed for compiling and running them. The Go programs can be either compiled to self-contained executable files with the go build command or run directly, using the go run command.

**Figure 1:**
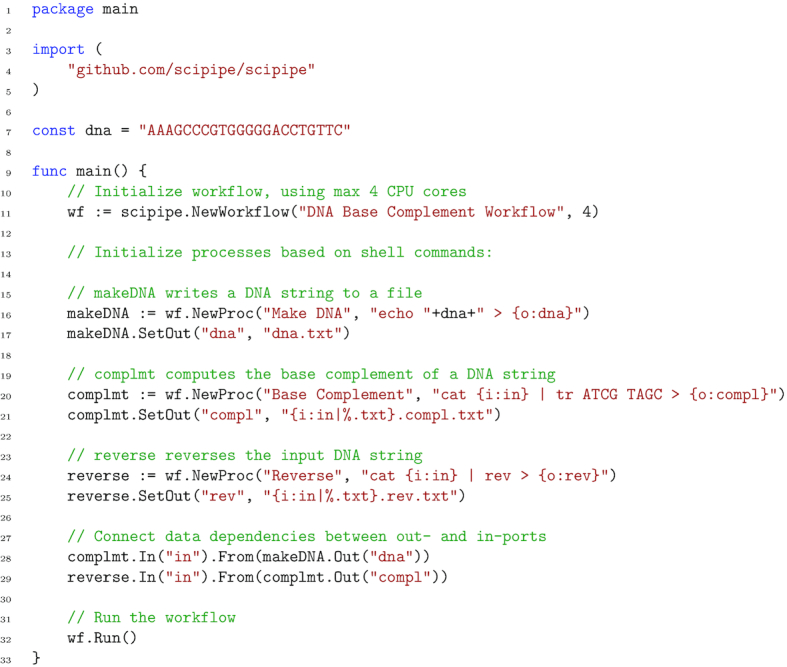
A simple example workflow implemented with SciPipe. The workflow computes the reverse base complement of a string of DNA, using standard UNIX tools. The workflow is a Go program and is supposed to be saved in a file with the .go extension and executed with the go run command. On line 4, the SciPipe library is imported, to be later accessed as scipipe. On line 7, a short string of DNA is defined. On lines 9–33, the full workflow is implemented in the program’s main() function, meaning that it will be executed when the resulting program is executed. On line 11, a new workflow object (or “struct” in Go terms) is initiated with a name and the maximum number of cores to use. On lines 15–25, the workflow components, or "processes," are initiated, each with a name and a shell command pattern. Input file names are defined with a placeholder on the form {i:INPORTNAME} and outputs on the form {o:OUTPORTNAME}. The port-name will be used later to access the corresponding ports for setting up data dependencies. On line 16, a component that writes the previously defined DNA string to a file is initiated, and on line 17, the file path pattern for the out-port "dna" is defined (in this case a static file name). On line 20, a component that translates each DNA base to its complementary counterpart is initiated. On line 21, the file path pattern for its only out-port is defined. In this case, reusing the file path of the file it will receive on its in-port named "in," thus the {i:in} part. The %.txt part removes .txt from the input path. On line 24, a component that will reverse the DNA string is initiated. On lines 27–29, data dependencies are defined via the in- and out-ports defined earlier as part of the shell command patterns. On line 32, the workflow is being run. This code example is also available in the SciPipe source code repository Lampa [29].

The simplest way to write a SciPipe program is to write the workflow definition in the program’s main() function, which is executed when running the compiled executable file, or running the file as a script with go run. An example workflow written in this way is shown in Fig. 1 (also available in Lampa [29]), which provides a simple example workflow consisting of 3 processes, demonstrating a few of the basic features of SciPipe. The first process writes a string of DNA to a file, the second computes the base complement, and the last process reverses the string. All in all, the workflow computes the reverse base complement of the initial string.

As can be seen in Fig. 1 on line 11, a workflow object (or struct, in Go terminology) is first initialized, with a name and a setting for the maximum number of tasks to run at a time. Furthermore, on lines 15–25, processes are defined with the Workflow.NewProc() method on the workflow struct, with name and a command pattern that is very similar to the Bash shell command that would be used to run a command manually, but where concrete file names have been replaced with placeholders, in the form {i:INPORTNAME}, {o:OUTPORTNAME}, or {p:PARAMETERNAME}. These placeholders define input and output files, as well as parameter values, and work as a sort of templates, which will be replaced with concrete values as concrete tasks are scheduled and executed.

As can be seen on lines 17, 21, and 25 (Fig. 1), output paths to use for output files are defined using the Process.SetOut() method, taking an out-port name and a pattern for how to generate the path. For simple workflows this can be just a static file name, but for more complex workflows with processes that produce >1 output on the same port—e.g., by processing different input files or using different sets of parameters—it is often best to reuse some of the input paths and parameter values configured earlier in the command pattern to generate a unique path for each output.

Finally, on lines 27–29, we see how in-ports and out-ports are connected in order to define the data dependencies between tasks. Here, the in-port and out-port names used in the placeholders in the command pattern described above are used to access the corresponding in-ports and out-ports and make connections between them, with a syntax in the general form of InPort.From(OutPort).

The last thing needed to do to run the workflow is seen on line 32, where the Workflow.Run() method is executed. Provided that the workflow code in Fig. 1 is saved in a file named workflow.go, it can be run using the go run command, like so:

$ go run workflow.go

This will then produce 3 output files and 1 accompanying audit log for each file, which can be seen by listing the files in a terminal:

dna.txt

dna.txt.audit.json

dna.compl.txt

dna.compl.txt.audit.json

dna.compl.rev.txt

dna.compl.rev.txt.audit.json

**Figure 2:**
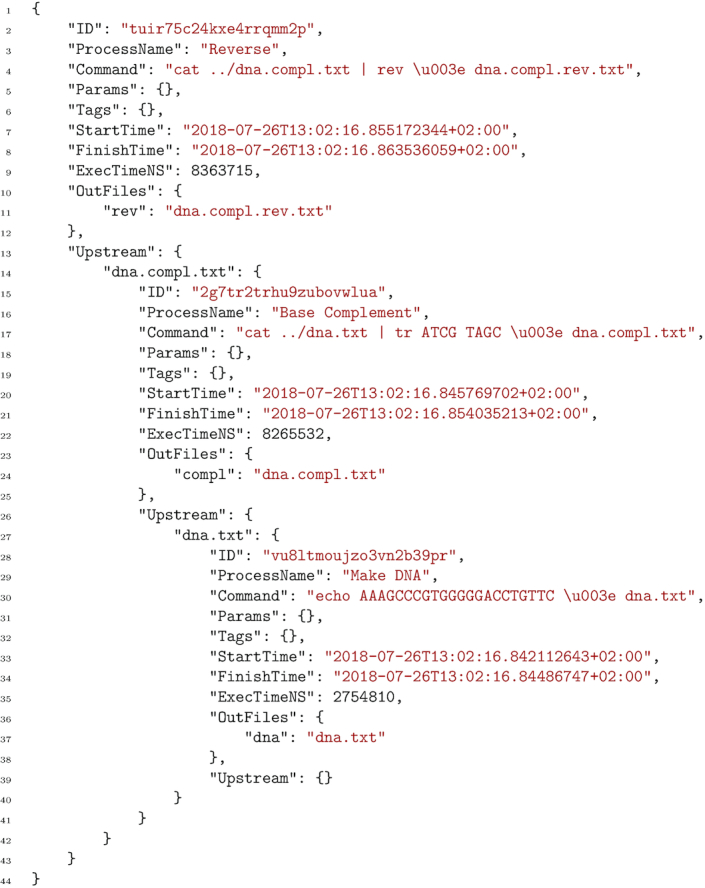
Example audit log file in JSON format [30] for a file produced by a SciPipe workflow. The workflow used to produce this audit log in particular is the one in Fig. 1. The audit information is hierarchical, with each level representing a step in the workflow. The first level contains metadata about the task executed last, to produce the output file that this audit log refers to. The field Upstream on each level contains a list of all tasks upstream of the current task, indexed by the file paths that each of the upstream tasks produced, and which was subsequently used by the current task. Each task is given a globally unique ID, which helps to deduplicate any duplicate occurrences of tasks when converting the log to other representations. Execution time is given in nanoseconds. Note that input paths in the command field are prepended with ../, compared to how they appear in the Upstream field. This is because each task is executed in a temporary directory created directly under the workflow’s main execution directory, meaning that to access existing data files, it has to first navigate up 1 step out of this temporary directory.

The file dna.txt should now contain the string AAAGCCCGTGGGGGACCTGTTC, and dna.compl.rev.txt should contain GAACAGGTCCCCCACGGGCTTT, which is the reverse base complement of the first string. In the last file above, the full audit log for this minimal workflow can be found. An example content of this file is shown in Fig. 2.

In this code example, it can be seen that both of the commands we executed are available and also that the "Reverse" process lists its ”upstream” processes, which are indexed by the input file names in its command. Thus, under the dna.compl.txt input file, we find the "Base Complement" process together with its metadata, and 1 further upstream process (the "Make DNA" process). This hierarchic structure of the audit log ensures that the complete audit trace, including all commands contributing to the production of an output file, is available for each output file of the workflow.

More information about how to write workflows with SciPipe is available on the documentation website [31]. Note that the full documentation on this website is also available in a folder named docs inside the SciPipe Git repository, which ensures that documentation for the version currently used is always available.

### Dynamic scheduling

Because SciPipe is built on the principles from FBP (see the Methods section for more details), a SciPipe program consists of independently and concurrently running processes, which schedule new tasks continually during the workflow run. This is here referred to as "dynamic scheduling." This means that it is possible to create a process that obtains a value and passes it on to a downstream process as a parameter, so that new tasks can be scheduled with it. This feature is important in machine learning workflows, where hyper parameter tuning is often used to find an optimal value of a parameter, such as cost for support vector machines, which is then used to parametrize the final training part of the workflow.

### Reusable components

**Figure 3:**
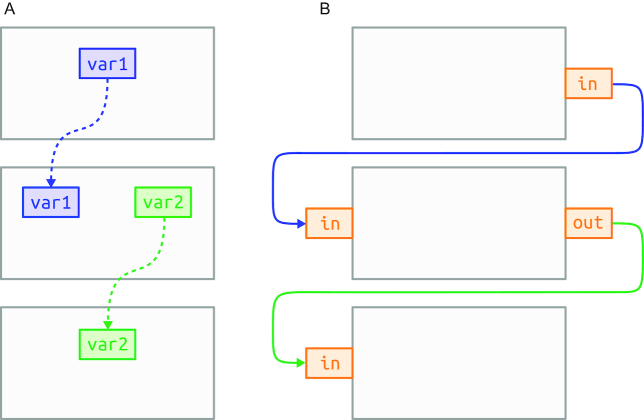
Comparison between dataflow variables and FBP ports in terms of dependency definition. A, How dataflow variables (blue and green) shared between processes (in grey) make the processes tightly coupled. In other words, process and network definitions get intermixed. B, How ports (in orange) bound to processes in FBP allow the network definition to be kept separate from process definitions. This enables processes to be reconnected freely without changing their internals.

Based on principles from FBP, the workflow graph in SciPipe is defined by making connections between port objects bound to processes. This enables the dependency graph definition to be kept separate from the process definitions. This is in contrast to other ways of connecting dataflow processes, such as with dataflow variables, which are shared between process definitions. This makes processes in FBP fully self-contained, meaning that libraries of reusable components can be created and that components can be loaded on-demand when creating new workflows. A graphical comparison between dependencies defined with dataflow variables and FBP ports is shown in Fig. 3.

### Running subsets of workflows

With pull-based workflow tools such as Snakemake or Luigi, it is easy to reproduce a particular output file on-demand because the scheduling mechanism is optimized for the use case of asking for a specific file and calculating all the tasks required to be executed based on that.

With push-based workflow tools, however, reproducing a specific set of files without running the full workflow is not always straightforward. This is a natural consequence of the push-based scheduling strategy, and dataflow in particular, because the identities and quantities of output files might not be known before the workflow is run.

SciPipe provides a mechanism for partly solving this lack of “on-demand file generation” in push-based dataflow tools, by allowing all files of a specified process to be reproduced on-demand. That is, the user can tell the workflow to run all processes in the workflow upstream of, and including, a specified process, while skipping processes downstream of it.

This has turned out to be very useful when iteratively refactoring or developing new pipelines. When a part in the middle of a long sequence of processes need to be changed, it is helpful to be able to test-run the workflow up to that particular process only, not the whole workflow, to speed up the development iteration cycle.

### Other characteristics

Below are a few technical characteristics and considerations that are not necessarily unique to SciPipe but could be of interest to potential users assessing whether SciPipe fits their use cases.

#### Data-centric audit log

The audit log feature in SciPipe collects metadata about every executed task (concrete shell command invocation), which is passed along with every file that is processed in the workflow. It writes a file in the ubiquitous JSON format, with the full trace of tasks executed for every output in the workflow, with the same name as the output file in question but with the additional file extension .audit.json. Thus, for every output in the workflow, it is possible to check the full record of shell commands used to produce it. An example audit log file can be seen in Fig. 2.

This data-oriented provenance reporting contrasts with provenance reports common in many workflow tools, which often provide 1 report per workflow run only, meaning that the link between data and provenance report is not as direct.

The audit log feature in SciPipe in many aspects reflects the recommendations by Gil and Garijo [32] for developing provenance reporting in workflows, such as producing a coherent, accurate, inspectable record for every output data item from the workflow. By producing provenance records for each data output rather than for the full workflow only, SciPipe could provide a basis for the problem of iteratively developing workflow variants, as outlined by Carvalho et al. [33].

SciPipe also loads any existing provenance reports for existing files that it uses, and merges these with the provenance information from its own execution. This means that even if a chain of processing is spread over multiple SciPipe workflow scripts and executed at different times by different users, the full provenance record is still being kept and assembled, as long as all workflow steps were executed using SciPipe shell command processes. The main limitation to this “follow the data” approach is for data generated externally to the workflow or by SciPipe components implemented in Go. For external processes, it is up to the external process to generate any reporting. For Go-based components in SciPipe, these cannot currently dump a textual version of the Go code executed. This constitutes an area of future development.

**Figure 4:**
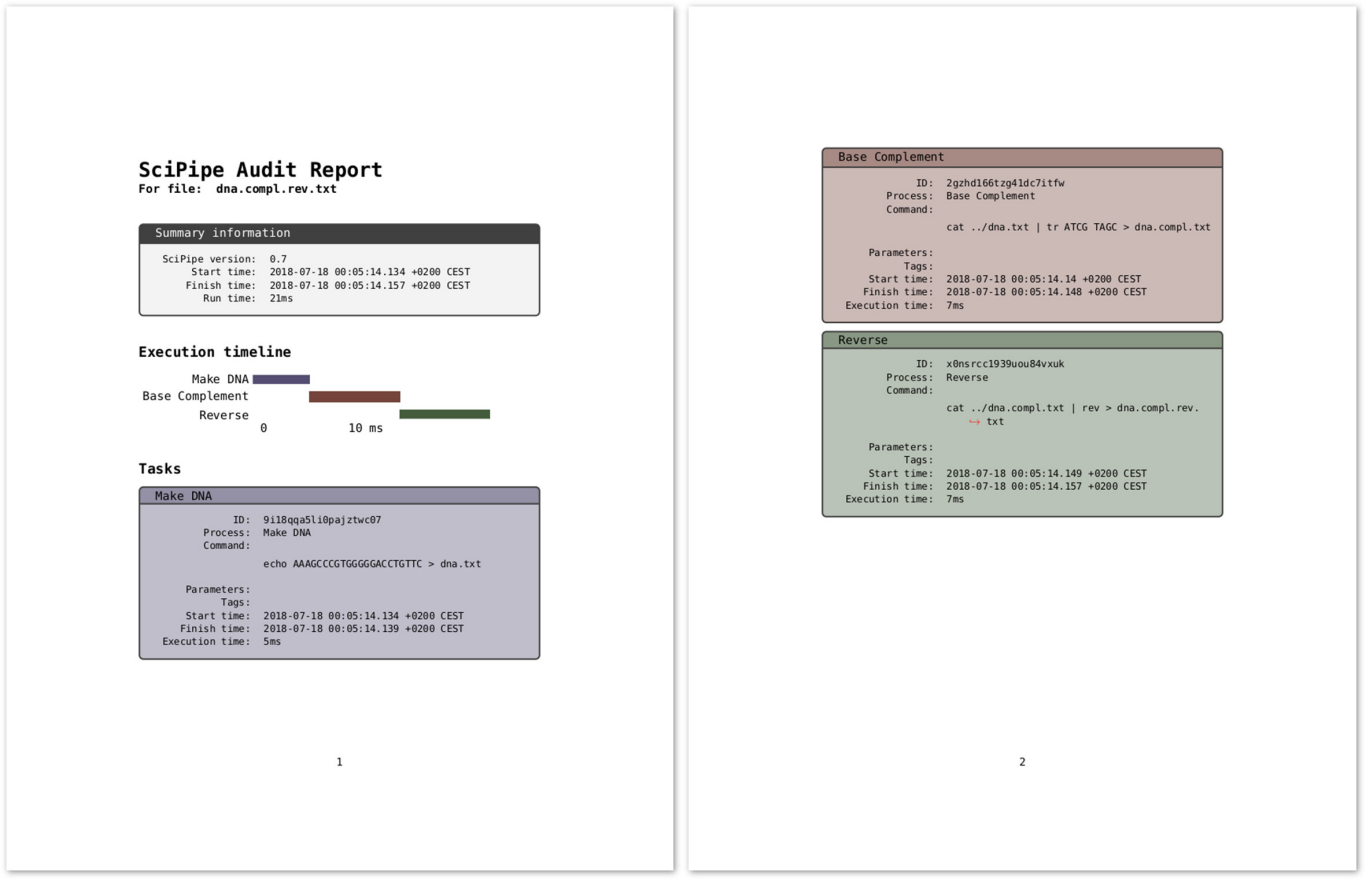
Audit report for the last file generated by the code example in Fig. 1, converted to TeX with SciPipe’s experimental audit2tex feature and then converted to PDF with pdfTeX. At the top, the PDF file includes summary information about the SciPipe version used and the total execution time. After this follows an execution timeline, in a Gantt chart style, that shows the relative execution times of individual tasks in a graphical manner. After this follows a comprehensive list of tables with information for each task executed towards producing the file to which the audit report belongs. The task boxes are color-coded and ordered in the same way that the tasks appear in the timeline.

SciPipe provides experimental support for converting the JSON structure into reports in HTML and TeX format, or into executable Bash scripts that can reproduce the file that the audit report describes from available inputs or from scratch. These tools are available in the scipipe helper command. The TeX report can be easily further converted to PDF using the pdflatex command of the pdfTex software [34]. An example of such a PDF report is shown in Fig. 4, which was generated from the audit report for the last file generated by the code example in Fig. 1.

Note that the JSON format used natively by SciPipe is not focused on adhering to a standard such as the W3C recommended standard for provenance information, W3C PROV [35]. To follow the approach taken with the scipipe helper tool, support for W3C PROV serialized, e.g., to JSON-LD [36] would be a most suitable additional conversion target and is planned for future development.

Note also that the provenance log in SciPipe can be seen as complementary to more technically comprehensive but also more low-level approaches to reproducibility such as ReproZip [37]. ReproZip monitors not just shell command executions but every system call made during a workflow run to enable all required dependencies to be captured and packed into a reproducible archive. One way to contrast these 2 approaches is that while the primary goal of ReproZip is reproducible execution, the provenance report in SciPipe serves multiple purposes, where understandability of executed analyses has a much more pronounced role.

#### Atomic writes

SciPipe ensures that cancelled workflow runs do not result in half-written output files being mistaken for finished ones. It does this by executing each task in a temporary folder and moving all newly created files into their final location after the task is finished. By using a folder for the execution, any extra files created by a tool that are not explicitly configured by the workflow system are captured and treated in an atomic way. Examples of where this is needed is for the 5 extra files created by bwa index [38] when indexing a reference genome in FASTA format.

#### Streaming support

In data-intensive fields such as next-generation sequencing, it is common for intermediate steps of pipelines to produce large amounts of data, often multiplying the storage requirements considerably compared to the raw data from sequencing machines [39]. To help ease these storage requirements, SciPipe provides the ability to optionally stream data between 2 tasks via random access memory (RAM) instead of saving to disk between task executions. This approach has 2 benefits. First, the data do not need to be stored on disk, which can lessen the storage requirements considerably. Second, it enables the downstream task to start processing the data from the upstream task immediately as soon as the first task has started to produce partial output. It thus enables pipeline parallelism to be achieved in addition to data parallelism and can thereby shorten the total execution time of the pipeline.

#### Flexible file naming and data “caching”

SciPipe allows flexible naming of the file path of every intermediate output in the workflow, based on input file names and parameter values passed to a process. This enables the creation of meaningful file-naming schemes, to make it easy to manually explore and sanity-check outputs from workflows.

Configuring a custom file-naming scheme is not required, however. If no file path is configured, SciPipe will automatically create a file path that ensures that 2 tasks with different parameters or input data will never clash and that 2 tasks with the same command signature, parameters, and input files will reuse the same cached data.

### Usage

We have successfully used SciPipe to train machine learning models for off-target binding profiles for early hazard detection of candidate drug molecules in early drug discovery [40]. This study was run on a single HPC node on the Rackham cluster [41] at UPPMAX HPC center at Uppsala University, which has 2 central processing units (CPUs) with 10 physical and 20 virtual cores each, and 128–256 GB of RAM (we had access to nodes with different amounts of RAM and were using whatever our application needed).

Owing to some changes in how we performed the training we did not get the opportunity to try out the exact same situation that we were struggling with with SciLuigi before, but based on experiments done on compute nodes on the same HPC cluster, it has been verified that SciPipe is able to handle up to 4,999 concurrent idle shell commands (which could be, e.g., monitoring jobs on the SLURM resource manager [42]), as opposed to the maximum of ∼64 concurrent commands with SciLuigi.

Apart from that and the occasional workflow in the wild [43], the SciPipe library has not yet seen much adoption outside our research group. Based on the high interest for the library on GitHub (486 stars and 36 forks at the time of writing), we think this is just a matter of time. We also think the interest in workflows implemented in compiled languages will increase as data sets continue to increase in size and performance and robustness issues grow more and more important. Because SciPipe is a somewhat more complex tool than the most popular ones such as Galaxy, we are planning to produce more tutorial material such as videos and blog posts to help newcomers get started.

### Known limitations

Below we list a number of known limitations of SciPipe that might affect the decision of whether to use SciPipe for a particular use case.

First, the fact that writing SciPipe workflows requires some basic knowledge of the Go programming language can be off-putting to users who are not well-acquainted with programming. Go code, although it takes inspiration from scripting languages, is still markedly more verbose and low-level in nature than Python and can take a little longer to get used to.

Second, the level of integration with HPC resource managers is currently quite basic compared to some other workflow tools. The SLURM resource manager can readily be used by using the Prepend field on processes to add a string with a call to the salloc SLURM command, but more advanced HPC integration is planned to be addressed in upcoming versions.

Third, the way commands are defined in SciPipe is quite simplistic compared to some other approaches. Approaches such as the CWL tool description format [44] and the Boutiques [45] framework provide more semantically comprehensive description of the command-line format. Boutiques also provides certain features for validation of inputs, which can help avoid connecting workflow inputs and outputs that are not compatible. We see this as an exciting area for future development, and where community contributions to the open source code will be especially welcome.

Furthermore, reproducing specific output files is not as natural and easy as with pull-based tools like Snakemake, although SciPipe provides a mechanism to partly resolve this problem.

Finally, SciPipe does not yet support integration with the CWL [19] for interoperability of workflows, and with the W3C PROV [35] format for provenance information. These are high-priority areas for future developments.

## Case Studies

To demonstrate the usefulness of SciPipe, we have used it to implement a number of representative pipelines from drug discovery and bioinformatics with different characteristics and hence requirements on the workflow system. These workflows are available in a dedicated git repository on GitHub [46].

### Machine learning pipeline in drug discovery

The initial motivation for building SciPipe stemmed from problems encountered with complex dynamic workflows in machine learning for drug discovery applications. It was thus quite natural to implement an example of such a workflow in SciPipe. To this end we re-implemented a workflow implemented previously for the SciLuigi library [26], which was itself based on an earlier study [47].

**Figure 5:**
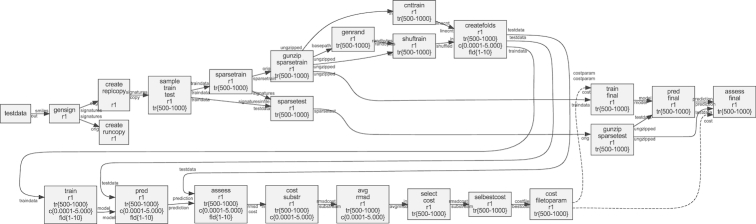
Directed graph of the machine learning in drug discovery case study workflow, plotted with SciPipe’s workflow plotting function. The graph has been modified for clarity by collapsing the individual branches of the parameter sweeps and cross-validation fold generation. The layout has also been manually made more compact to be viewable in print. The collapsed branches are indicated by intervals in the box labels. tr{500-8000} represent branching into training data set sizes 500, 1,000, 2,000, 4,000, 8,000. c{0.0001-5.0000} represent cost values 0.0001, 0.0005, 0.001, 0.005, 0.01, 0.05, 0.1, 0.25, 0.5, 0.75, 1, 2, 3, 4, and 5, while fld{1-10} represent cross-validation folds 1−10. Nodes represent processes, while edges represent data dependencies. The labels on the edge heads and tails represent ports. Solid lines represent data dependencies via files, while dashed lines represent data dependencies via parameters, which are not persisted to file, only transmitted via RAM.

In short, this workflow trains predictive models using the LIBLINEAR software [48] with molecules represented by the signature descriptor [49]. For linear support vector machines a cost parameter needs to be tuned, and we tested 15 values (0.0001, 0.0005, 0.001, 0.005, 0.01, 0.05, 0.1, 0.25, 0.5, 0.75, 1, 2, 3, 4, 5) in a 10-fold cross-validated parameter sweep. Five different training set sizes (500, 1,000, 2,000, 4,000, 8,000) were tested and evaluated with a test set size of 1,000. The raw data set consists of 10,000 logarithmic solubility values chosen randomly from a data set extracted from PubChem [50] according to details in Lampa et al. [26]. The workflow is schematically shown in Fig. 5 and was plotted using SciPipe’s built-in plotting function. The figure has been modified for clarity by collapsing the individual branches of the parameter sweeps and cross-validation folds, as well as by manually making the layout more compact.

The implementation in SciPipe was done by creating components that are defined in separate files (named comp_COMPONENTNAME in the repository), which can thus be reused in other workflows. This shows how SciPipe can be used to create workflows based on reusable, externally defined components.

The fact that SciPipe supports parametrization of workflow steps with values obtained during the workflow run meant that the full workflow could be kept in a single workflow definition, in 1 file. This also made it possible to create audit logs for the full workflow execution for the final output files and to create the automatically plotted workflow graph shown in Fig. 5. This is in contrast to the SciLuigi implementation, where the parameter sweep to find the optimal cost, and the final training, had to be implemented in separate workflow files (wffindcost.py and wfmm.py in [51]) and executed as a large number of completely separate workflow runs (1 for each data set size), which meant that logging became fragmented into a large number of disparate log files.

### Genomics cancer analysis pipeline

**Figure 6:**
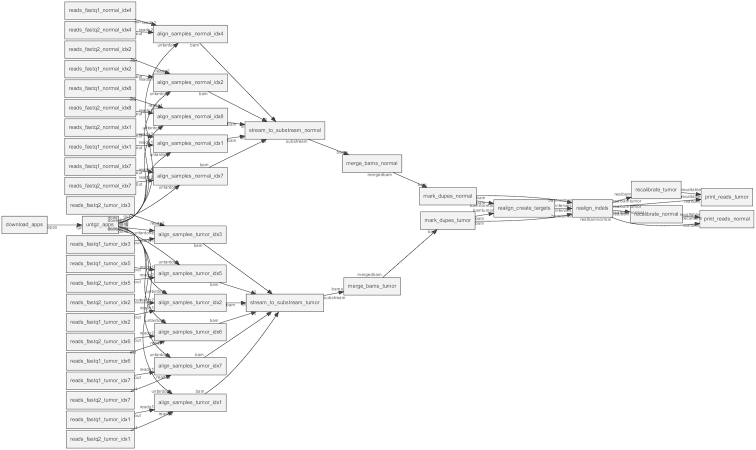
Directed graph of workflow processes in the Genomics/Cancer Analysis pre-processing pipeline, plotted with SciPipe’s workflow plotting function. Nodes represent processes, while edges represent data dependencies. The labels on the edge heads and tails represent ports.

Sarek [52] is an open source analysis pipeline to detect germline or somatic variants from whole-genome sequencing, developed by the National Genomics Infrastructure and National Bioinformatics Infrastructure Sweden, which are both platforms at Science for Life Laboratory. To test and demonstrate the applicability of SciPipe to genomics use cases the pre-processing part of the Sarek pipeline was implemented in SciPipe. See Fig. 6 for a directed process graph of the workflow, plotted with SciPipe’s workflow plotting function.

The test data in the test workflow consists of multiple samples of normal and tumour pairs. The workflow starts with aligning each sample to a reference genome using BWA [38] and forwarding the results to Samtools [53], which saves the result as a sorted BAM file. After each sample has been aligned, Samtools is again used, to merge the normal and tumour samples into 1 BAM [53] file for tumour samples and 1 for normal samples. Picard [54] is then used to mark duplicate reads in both the normal and tumour sample BAM files, whereafter GATK [55] is used to recalibrate the quality scores of all reads. The outcome of the workflow is 2 BAM files: 1 containing all the normal samples and 1 containing all the tumour samples.

Genomics tools and pipelines have their own set of requirements, which was shown by the fact that some aspects of SciPipe had to be modified to ease development of this pipeline. In particular, many genomics tools produce additional output files apart from those specified on the command line. One example of this is the extra files produced by BWA when indexing a reference genome in FASTA format. The bwa index command produces some 5 files that are not explicitly defined on the command line (with the extensions .bwt, .pac, .ann, .amb, and .sa). Based on this realization, SciPipe was amended with a folder-based execution mechanism that executes each task in a temporary folder, which keeps all output files separate from the main output directory until the whole task has completed. This ensures that files that are not explicitly defined and handled by SciPipe are also captured and handled in an atomic manner, so that finished and unfinished output files are always properly separated.

Furthermore, agile development of genomic tools often requires being able to see the full command that is used to execute a tool, because of the many options that are available to many bioinformatics tools. This workflow was thus implemented with ad hoc commands, which are defined in-line in the workflow. The ability to do this shows that SciPipe supports different ways of defining components, depending on what fits the use case best.

The successful implementation of this genomics pipeline in SciPipe thus both ensures and shows that SciPipe works well for tools common in genomics.

### RNA-seq/transcriptomics pipeline

To test the ability of SciPipe to work with software used in transcriptomics, some of the initial steps of a generic RNA-sequencing workflow were also implemented in SciPipe. Common steps that are needed in transcriptomics are to run quality controls and generate reports of the analysis steps.

The RNA-sequencing case study pipeline implemented for this article uses FastQC [56] to evaluate the quality of the raw data being used in the analysis before aligning the data using STAR [57]. After the alignment is done, it is evaluated using QualiMap [58], while the Subread package [59] is used to do a feature counting.

**Figure 7:**
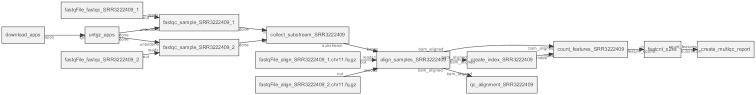
Directed graph of workflow processes in the RNA-Seq Pre-processing workflow, plotted with SciPipe’s workflow plotting function. Nodes represent processes, while edges represent data dependencies. The labels on the edge heads and tails represent ports.

The final step of the workflow is to combine all the previous steps for a composite analysis using MultiQC [60], which will summarize the quality of both the raw data and the result of the alignment into a single quality report. See Fig. 7 for a directed process graph of the workflow, plotted with SciPipe’s workflow plotting function.

The successful implementation of this transcriptomics workflow in SciPipe ensures that SciPipe works well for different types of bioinformatics workflows and is not limited to 1 specific sub-field of bioinformatics.

### Conclusions

SciPipe is a programming library that provides a way to write complex and dynamic pipelines in bioinformatics, cheminformatics, and more generally in data science, and machine learning pipelines involving command-line applications.

Dynamic scheduling allows new tasks to be parametrized with values obtained during the workflow run, and the FBP principles of separate network definition and named ports allow the creation of a library of reusable components. By having access to the full power of the Go programming language to define workflows, existing tooling is leveraged.

SciPipe adopts state-of-the-art strategies for achieving atomic writes, caching of intermediate files, and a data-centric audit log feature that allows identification of the full execution trace for each output, which can be exported into either human-readable HTML or TeX/PDF formats or executable Bash scripts.

SciPipe also provides some features not commonly found in many tools such as support for streaming via Unix named pipes, the ability to run push-based workflows up to a specific stage of the workflow, and flexible support for file naming of intermediate data files generated by workflows. SciPipe workflows can also be compiled into stand-alone executables, making deployment of pipelines maximally easy, requiring only the presence of Bash and any external command-line tools used on the target machine.

The fact that SciPipe is a small library without required external dependencies apart from the Go tool chain and Bash means it is expected to be possible to be maintained and developed in the future even without a large team or organization backing it.

The applicability of SciPipe for cheminformatics, genomics, and transcriptomics pipelines has been demonstrated with case study workflows in these fields.

## Methods

### The Go programming language

The Go programming language (referred to hereafter as ”Go”) was developed by Robert Griesemer, Rob Pike, and Ken Thompson at Google to provide a statically typed and compiled language that makes it easier to build highly concurrent programs that can also make good use of multiple CPU cores (i.e., “parallel program”) in contrast to what is the case in widespread compiled languages like C++ [61]. It tries to provide this by providing a small, simple language, with concurrency primitives—go-routines and channels—built into the language. Go-routines, which are so-called lightweight threads, are automatically mapped, or multiplexed, onto physical threads in the operating system. This means that very large numbers of go-routines can be created while maintaining a low number of operating system threads, such as 1 per CPU core on the computer at hand. This makes Go an ideal choice for problems where many asynchronously running processes need to be handled concurrently, and for making efficient use of multi-core CPUs.

The Go compiler statically links all its code as part of the compilation. This means that all dependent libraries are compiled into the executable file. Because of this, SciPipe workflows can be compiled into self-contained executable files without external dependencies apart from the Bash shell and any external command-line tools used by the workflow. This makes deploying Go programs (and SciPipe workflows) to production very easy.

Go programs are high-performing, often an order of magnitude faster than interpreted languages like Python, and on the same order of magnitude as the fastest languages, such as C, C++, and Java [62].

### Dataflow and FBP

Dataflow is a programming paradigm oriented around the idea of independent, asynchronously running processes that only talk to each other by passing data between each other. This data passing can happen in different ways, such as via dataflow variables or via first-in-first-out channels.

FBP [63] is a paradigm for programming developed by John Paul Morrison at IBM in the late 60s/early 70s to provide a composable way to assemble programs to be run on mainframe computers by customers such as large banks. It is a specialized version of dataflow, adding the ideas of separate network definition, named ports, channels with bounded buffers, and information packets (representing the data) with defined lifetimes. Just as in dataflow, the idea is to divide a program into independent processing units called “processes,” which are allowed to communicate with the outside world and other processes solely via message passing. In FBP, this is always done over channels with bounded buffers, which are connected to named ports on the processes. Importantly, the network of processes and channels is in FBP described “separately” from the process implementations, meaning that the network of processes and channels can be reconnected freely without changing the internals of processes.

This strict separation of the processes, the separation of network structure from processing units, and the loosely coupled nature of its only way of communication with the outside world (message passing over channels) makes flow-based programs extremely composable and naturally component-oriented. Any process can always be replaced with any other process that supports the same format of the information packets on its in-ports and out-ports.

Furthermore, because the processes run asynchronously, FBP is, just like Go, very well-suited to make efficient use of multi-core CPUs, where each processing unit can suitably be placed in its own thread or co-routine to spread out on the available CPU cores on the computer. FBP has a natural connection to workflow systems, where the computing network in an FBP program can be likened to the network of dependencies between data and processing components in a workflow [26]. SciPipe leverages the principles of separate network definition and named ports on processes. SciPipe has also taken some inspiration for its API design from the GoFlow [64] Go-based FBP framework.

## Availability of supporting source code and requirements

- Project name: SciPipe (SciPipe, RRID:SCR_017086)
- Documentation and project home page: http://scipipe.org [31]
- Source code repository: https://github.com/scipipe/scipipe [27]
- Persistent source code archive: https://doi.org/10.5281/zenodo.1157941 [28]
- Operating system(s): Linux, Unix, Mac
- Other requirements: Go 1.9 or later, Bash, GraphViz (for workflow graph plotting), LaTeX (for PDF generation)
- License: MIT

## Availability of supporting data and materials

- Source code for case study workflows: https://github.com/pharmbio/scipipe-demo [46]
- The raw data for the machine learning in drug discovery demonstration pipeline are available at: https://doi.org/10.5281/zenodo.1324443 [65]
- The applications for the machine learning in drug discovery case study are available at: https://doi.org/10.6084/m9.figshare.3985674 [66]
- The raw data and tools for the genomics and transcriptomics workflows are available at: https://doi.org/10.5281/zenodo.1324425 [67]

## Abbreviations

API: application programming interface; CPU: central processing unit; CWL: Common Workflow Language; DSL: domain-specific language; FBP: flow-based programming; HDFS: Hadoop distributed file system; HPC: high-performance computing; JSON: JavaScript Object Notation; RAM: random access memory.

## Competing interests

The authors declare that they have no competing interests.

## Funding

This work has been supported by the Swedish strategic research programme eSSENCE, the Swedish e-Science Research Centre (SeRC), National Bioinformatics Infrastructure Sweden (NBIS), and the European Union’s Horizon 2020 research and innovation programme under grant agreement No. 654241 for the PhenoMeNal project.

### Authors’ contributions

O.S. and S.L. conceived the project and the idea of component-based workflow design. S.L. came up with the idea of using Go and FBP principles to implement a workflow library, designed and implemented the SciPipe library, and implemented case study workflows. M.D. contributed to the API design and implemented case study workflows. J.A. implemented the TeX/PDF reporting function and contributed to case study workflows. O.S. supervised the project. All authors read and approved the manuscript.

## Supplementary Material

GIGA-D-18-00390_Original_Submission.pdf

GIGA-D-18-00390_Revision_1.pdf

GIGA-D-18-00390_Revision_2.pdf

Response_to_Reviewer_Comments_Original_Submission.pdf

Reviewer_1_Report_Original_Submission -- Gregory Kiar 10/24/2018 Reviewed

Reviewer_1_Report_Revision_1 -- Gregory Kiar 3/20/2019 Reviewed

Reviewer_2_Report_Original_Submission -- Konstantinos Krampis, PhD 11/2/2018 Reviewed

Reviewer_3_Report_Original_Submission -- Lars Ailo Bongo 11/3/2018 Reviewed
