# Supplementary material for: SciPipe: A workflow library for agile development of complex and dynamic bioinformatics pipelines
Source: Gigascience. 2019 Apr 26;8(5):giz044. doi: 10.1093/gigascience/giz044 (PMC6486472; doi:10.1093/gigascience/giz044)
Supplement: Response_to_Reviewer_Comments_Original_Submission.pdf [file giz044_response_to_reviewer_comments_original_submission.pdf]

Author's Response To Reviewer Comments

Close

Dear Editor,

We were pleased to read the many positive and constructive comments from all the three reviewers; there were several useful suggestions which has improved the manuscript. We have updated the manuscript accordingly and provide below point-by-point responses to the reviewers' comments. With these improvements, we hope You will find it acceptable for publication in GigaScience.

Sincerely,

Samuel Lampa and Co-authors

Reviewer #1

Reviewer #1: The submitted paper presents a workflow engine for scientific computing, with particular emphasis on applications in genomics, bioinformatics, and transcriptomics. The authors do a nice job at emphasizing the usefulness of their particular tool, highlighting limitations of prior art, demonstrating novel features in Scipipe, and suggesting design principles which are useful for others in the space of workflow engine development. The presented tool is written in the elegant and popular Go Programming Language, and proved easy to use for even the Go-novice that is this reviewer. The manuscript is very well written, and was easy to follow.

While I have attached notes and comments below, the only area I feel needs to be addressed which has significant impact the quality and usefulness of this manuscript and tool is that of interoperability. The authors discuss other standards or engines in this area, and while they mention the plan for future integration with the Common Workflow Language, they do not discuss the integration with or adoption of other standards. As there are many workflow engines, and several dominant options in the space of bioinformatics such as Galaxy, while Scipipe may be preferable in some ways the cost for authors to switch is
non-zero. What is the motivation for scientists who have their workflows integrated in one of these other systems to switch? What tools are there or will there be to aid in this process? These are questions which readers and potential users may be thinking, and I believe are important to address.
Regarding interoperability, there are also various standards and tools which exist in other spaces covered here. For instance, there are tools which record or ensure interoperability of provenance records, such as Reprozip for managing file I/O provenance and constructing access graphs of executions, and W3C-PROV for representing records as disambiguated entities. This reviewer also found the representation of command-lines themselves was rather simple, without any type-checking of parameters (which, while not performed directly in Bash by command-line applications, can be of use to prevent connecting nodes which may be incompatible, such as a string output being connected to an input expecting a number, let alone bounds on reasonable values for it), whereas standards exist such as Boutiques that address this for command-line utilities through the use of JSON tool descriptors and utilities which aid in the validation of parameters. Similarly, a common workflow engine in neuroinformatics, Nipype, exists as a very similar tool to Scipipe but has been written in Python with neuroscience applications in mind, though it is in principle also agnostic to domain. In each of these cases, it would be valuable to consider adopting established standards where possible - or import/export functionality where this isn't possible - and justify the decisions made in Scipipe in their context. While Scipipe presents a novel workflow management system, addressing the above points and interoperability between other frameworks may put to rest any concerns in adopting Scipipe or integrating it within their current practices.

Authors' response

First, thank you for the kind words!

In answer to the raised concerns, we do not see SciPipe as a silver bullet to replace existing successful systems, but rather as a solution to those researchers and developers facing particularly complex workflow challenges - increasingly common because of the upsurge in machine learning and cross-validation - which might motivate to trade some ease-of-use for the required flexibility to be able to model their computations at all. We thus think the selection of primary target group for SciPipe in itself will ensure the motivation for switching is there.

We do have plans for improved interoperability though, and want to do this primarily as subcommands added to the scipipe helper tool, to avoid cluttering the core library with non-essential functionality. The reason for this is that one core design goal of SciPipe has been to retaining a simple implementation to keep it maintainable with minimal efforts in the long run.

We are thankful for the additional pointers about standards and formats, and have updated the manuscripts brief reviews of these tools, and our plans and views regarding them.

Reviewer #1

Below are my notes on specific sections of the paper which questions or comments. There is some overlap with the above paragraphs as I have tried to identify where I believe some of these points could be well addressed.

Page 1, Line 51, column 2
- Is the implication that Bash, Python, or Perl are more prone to becoming fragile than Go? Is this the case? If so, why?

Authors' response

The comparison (which also follows in the next paragraph) is not made with Go per se, but with workflow tools or libraries. So, the comparison stands between “plain scripts” and workflow tools or libraries, which could sometimes be implemented in scripting languages.

We have reworded the sentence slightly to clarify this intention, as well as removed the paragraph split just after that sentence, to clarify that the explanation follows right after it.

Reviewer #1

Page 2 & 3
- The authors did a very nice and thorough review of many tools in the space of workflow management tools. An alternative that wasn't mentioned here was Nipype, a tool commonly used in neuroscience for workflow management, though in principle it is domain agnostic. In particular, I notice that many of the features described as desired here, including branching, provenance tracking, and enabling reproducible computation, having both a command-line and in-language API, etc., are very similar to those of Nipype. I would like the authors to do a review and comparison of these tools, as well. Another tool or representation of potential interest could be the Common Workflow Language, which I only found brief mention of later on.

Authors' response

Thanks for the suggestion of Nipype. Because of the specific focus on the Neuroimaging community, we had not noticed the generality of Nipype before. Upon reading the paper, we indeed see that it shares many similarities with SciPipe. We have added it to the comparison of existing tools. Regarding CWL, we note that CWL is not really a workflow implementation, but rather a language, with multiple implementations. The language is what will be the limiting factor for what is easy to express though, so we have included a brief mention about it also in the introduction.

Reviewer #1

Page 3, Line 36, column 1
- Broken citation

Authors' response

Fixed, thanks!

Reviewer #1

Page 3, Line 48, column 1
- How does this provenance log compare to those obtained from Reprozip? The authors may wish to do a comparison of provenance standards in the first section, as well.

Authors' response

From our understanding, Reprozip is a complementary approach to SciPipe, which goes much deeper and traces and hijacks system calls to create a completely self-contained reproducible package, based on any tool, not just workflows. SciPipe provenance reports do not take this comprehensive technical approach, but rather gathers the semantics it does handle by each task invocation, and stores a trace of this information for every output from the workflow.

We have updated the manuscript with a bit of comparison to other provenance standards and tools, including W3C-PROV and Reprozip, both in the main text (W3C PROV and Reprozip) and under Known limitations (W3C PROV, Boutiques).

Reviewer #1

Figure 1
- What was the reason behind defining tasks in the way shown on lines 16 and 20? There are some standards, such as Boutiques (boutiques.github.io) and CWL which define task command-lines, including validating data typing, etc., that it seems could be of some use here to make sure that commands are being run meaningfully. For instance, these standards could perhaps enabling checking that all values in the DNA string are A, G, C, or T.
- I successfully re-executed this script after following the installation instructions found on the documentation page.

Authors' response

We have been evaluating the tool syntax tooling in CWL prior to developing our too (code here: https://github.com/NBISweden/workflow-tools-evaluation). Our choice to not prioritize any functionality based on this or similar standards has been an intentional decision because of the nontrivial amount of complexity that this type of parsing tooling typically adds to a tool. For example, the current SciPipe implementation is a completely self-contained Go library, without dependencies on other libraries apart from Bash and a Unix-like operating system.

We think it could be interesting to consider for future development though, in particular if it is possible to implement in the form of a subcommand in the stand-alone scipipe helper tool, as that would still enable to keep the core library (the part that is shipped with workflows) small and easy to maintain. We have updated the text to note this as interesting for future development.

Reviewer #1

Page 6, Line 37-47, column 1
- Are you defining this provenance data with respect to any accepted standard, such as JSON-LD (the

W3C-PROV compatible JSON format)? If not, how come, and what are the consequences of this custom definition of metadata?

Authors' response

The data we are storing here are a very straight-forward mapping from the internal data structures in SciPipe, to JSON. This is thus by definition the most compact and generic representation of the provenance data possible based on the data that we have. Our approach has then been to provide any other data formats (including HTML, TeX/PDF and executable Bash scripts) via converters in the scipipe commandline helper tool. We think this would be a very natural choice for how to implement conversion to W3C-PROV serialized as JSON-LD as well. We have updated the manuscript with a mention about this.

Reviewer #1

Remove commas:
- Page 4, Line 48, column 2: after "used"
- Page 6, Line 25, column 2: after "workflow system"
- Page 7, Line 31, column 1: after "programming language"

Authors' response

Fixed, thanks!

Reviewer #2

Reviewer #2: Excellent work, building a workflow system implementing the data flow paradigm for bioinformatics with a fast language (GO) as well. Some minor concerns I have are the following:

- This is obviously targeted to bioinformatics developers, but assuming there is a community that adopts it, would there be a way for someone who can do basic command line to use
it ? I am assuming that is there is a community many workflows will be published and a non developer could run it with a single command by just pointing to his / her datasets?

Authors' response

Thank you for kind words! Just as with Python scripts, Go code, and thus SciPipe workflows, can be made into very easy to use command-line programs, that can be used as easily as any other command-line program. Go additionally has the benefit that workflows can be compiled to self-contained executable files, so that the user does not even need to figure out how to execute a specific interpreter command, such as with Python, R or Perl.

Reviewer #2

- What other dependencies does it need besides GO to be installed ?

Authors' response

SciPipe requires a Unix or Linux like operating system, and the Bash shell. This information is available in the manuscript, under the section “Availability of supporting source code and requirements”

Reviewer #2

- Can it do parallelism in data chunks similar to Nextflow ? Many bioinformatics files (think for example

reads) can be processed in an embarrassingly parallel way, for example if they
are split and aligned to a reference genome.

Authors' response

SciPipe can indeed to parallel processing. SciPipe supports both so called embarrassingly parallel tasks, by enabling scatter/gather types of workflows. There is an example of this in the code repository: https://github.com/scipipe/scipipe/blob/master/examples/scatter_gather/scattergather.go

SciPipe also supports pipeline parallelism, firstly by means of processing multiple stages at once, as long as there are enough data to be travelling through the system that the first stages has work to do even when they have passed on some data to downstream stages. SciPipe additionally supports a features that as far as we know is not included even in Nextflow: Unix pipe-based streaming, using named pipes, to support pipeline parallelism across multiple stages even for single data items. Documentation for this feature is available here: http://scipipe.org/howtos/streaming/

Reviewer #2

- Finally, what about Docker containers? For example Nextflow has build in the option to pull containers "on the fly" with the tools preconfigured from repositories such as Dockstore etc
which have hundreds of pre-made containers. This is especially useful in the case of complex bioinformatics pipelines which have 10-15 different tools. Of course a developer can build a single container with all the tools pre-configured and run SciPipe from within this container, but if container support is available natively within the SciPipe implementation, developers can simply point to available (public or internally) containers with pre-configured tools, which will be started at runtime of the workflow in order to provide the algorithms which the workflow feeds the data
for processing during each different step.

Authors' response

SciPipe already supports running Singularity containers natively, by calling them like command-line programs. The path we have otherwise taken in regards to containers, is is to provide integration with Kubernetes. This is ongoing experimental work, available in a separate branch on GitHub.

Reviewer #2

Some corrections:
Line 33, right column better to use "Fig. 1" with bold letters.
Line 36, left column missing reference.
Line 46, right column, correct as "As can be seen on lines 17, 21 and 25, Fig.1", so that we know which figure we refer to.

Authors' response

Thanks! We have fixed the suggested corrections, except the first one, which we think violates the styling guidelines for the TeX template in use (we have not seen bold letter used in any other articles using the same template).

Reviewer #3

Reviewer #3: ##Comments to paper

This is a well written paper describing the, to my knowledge, first workflow manager implemented in Go. Although there are many alternative workflow managers, this work is motivated by the limitation of one of the state-of-the-art workflow managers (Luigi) that the authors have previously used and even

extended.

The paper describes the design of SciPipe, shows how it is used, and provides use cases. It does not provide any evaluation of SciPipe, nor does it describe system the use cases were run on. The latter should be included, since one of the motivations for SciPipe is the issues encountered with SciLuigi when run on more than 64 workers. The paper also does not describe how many users SciPipe has. Is it just used by the authors? I would also have liked a discussion about workflows, such as ADAM (https://github.com/bigdatagenomics/adam), that are implemented in Spark.

Authors' response

We have updated the paper with a “Usage” section, describing the known usage of SciPipe, including a brief discussion of the systems on which we have run real-world workflows.
We have also extended the introduction with a review of ADAM.

Reviewer #3

A minor issue: on page 3, line 36, there is a missing reference.

Authors' response

Thanks! Fixed.

Reviewer #3

Comments to the source code and documentation

The SciPipe webpage is well designed, with documentation and example workflows. The GitHub repository has 833 commits, with the last commit on August 18th. It has 426 stars and 27 forks, which suggest that there is interest in the community. The install documentation are a bit hard to find in the webpage, especially for someone that has not read the paper. There does not seem to be a test suite for SciPipe.

Authors' response

SciPipe does indeed include a test suite. The test suite in Go does not need to reside in a separate folder, but are put in files ending with “_test.go”. Tests are integrated and run in our continuous integration suite, and continuously updated coverage statistics are available at:
https://codecov.io/gh/scipipe/scipipe

Reviewer #3

I tested SciPipe on my laptop in Ubuntu on Windows. I have very limited knowledge of Go, so I just followed the examples on the webpage. They did work as described.

I first tested the RNA-seq case study. For it the documentation was less clear, and there were no instructions for how to do it. For example, how to specify the input dataset, which I later found was in the go code. The execution took a while, and it was hard to know if the program was working, or if it has crashed or waiting for input (especially since the first step downloads a 1.7GB file for which the size or progress is not shown). The case study failed, due to a missing library used by STAR. This is not a SciPipe issue, and it would not occur on a production system. SciPipe did however save the logs necessary to understand the issue.

Authors' response

We have now updated the individual case study workflow folders with simple README.md files, to help take out the guesswork. Thanks for pointing this out!

Reviewer #3

Second, I tested the drug discovery workflow. It could not be compiled due to:

./utils.go:45: t.Round undefined (type time.Duration has no field or method Round)

Authors' response

It turns out that this is caused by using a Go version below 1.9. We have added the version information in the dependencies listing in the manuscript.

Reviewer #3

Finally, I tested the genomics cancer workflow, which also failed due to a version issue in GenomeAnalysisTK.jar. Again, this is a third party installation error.

I did not do any more advanced testing of SciPipe, including using my own data, running it on more than one machine, nor stopping and restarting workflow execution.

Close
