# Supplementary material for: SciPipe: A workflow library for agile development of complex and dynamic bioinformatics pipelines
Source: Gigascience. 2019 Apr 26;8(5):giz044. doi: 10.1093/gigascience/giz044 (PMC6486472; doi:10.1093/gigascience/giz044)
Supplement: Reviewer_1_Report_Original_Submission -- Gregory Kiar — 10/24/2018 Reviewed [file giz044_reviewer_1_report_original_submission.pdf]

Reviewer Report

Title: SciPipe - A workflow library for agile development of complex and dynamic bioinformatics pipelines

Version: Original Submission **Date:** 10/24/2018

Reviewer name: Gregory Kiar

Reviewer Comments to Author:

The submitted paper presents a workflow engine for scientific computing, with particular emphasis on applications in genomics, bioinformatics, and transcriptomics. The authors do a nice job at emphasizing the usefulness of their particular tool, highlighting limitations of prior art, demonstrating novel features in Scipipe, and suggesting design principles which are useful for others in the space of workflow engine development. The presented tool is written in the elegant and popular Go Programming Language, and proved easy to use for even the Go-novice that is this reviewer. The manuscript is very well written, and was easy to follow.

While I have attached notes and comments below, the only area I feel needs to be addressed which has significant impact the quality and usefulness of this manuscript and tool is that of interoperability. The authors discuss other standards or engines in this area, and while they mention the plan for future integration with the Common Workflow Language, they do not discuss the integration with or adoption of other standards. As there are many workflow engines, and several dominant options in the space of bioinformatics such as Galaxy, while Scipipe may be preferable in some ways the cost for authors to switch is

non-zero. What is the motivation for scientists who have their workflows integrated in one of these other systems to switch? What tools are there or will there be to aid in this process? These are questions which readers and potential users may be thinking, and I believe are important to address.

Regarding interoperability, there are also various standards and tools which exist in other spaces covered here. For instance, there are tools which record or ensure interoperability of provenance records, such as Reprozip for managing file I/O provenance and constructing access graphs of executions, and W3C-PROV for representing records as disambiguated entities. This reviewer also found the representation of command-lines themselves was rather simple, without any type-checking of parameters (which, while not performed directly in Bash by command-line applications, can be of use to prevent connecting nodes which may be incompatible, such as a string output being connected to an input expecting a number, let alone bounds on reasonable values for it), whereas standards exist such as Boutiques that address this for command-line utilities through the use of JSON tool descriptors and utilities which aid in the validation of parameters. Similarly, a common workflow engine in neuroinformatics, Nipype, exists as a very similar tool to Scipipe but has been written in Python with neuroscience applications in mind, though it is in principle also agnostic to domain. In each of these cases, it would be valuable to consider adopting established standards where possible - or import/export functionality where this isn't possible - and justify the decisions made in Scipipe in their context. While Scipipe presents a novel workflow management system, addressing the above points and

interoperability between other frameworks may put to rest any concerns in adopting Scipipe or integrating it within their current practices.

Below are my notes on specific sections of the paper which questions or comments. There is some overlap with the above paragraphs as I have tried to identify where I believe some of these points could be well addressed.

Page 1, Line 51, column 2

- Is the implication that Bash, Python, or Perl are more prone to becoming fragile than Go? Is this the case? If so, why?

Page 2 & 3

- The authors did a very nice and thorough review of many tools in the space of workflow management tools. An alternative that wasn't mentioned here was Nipype, a tool commonly used in neuroscience for workflow management, though in principle it is domain agnostic. In particular, I notice that many of the features described as desired here, including branching, provenance tracking, and enabling reproducible computation, having both a command-line and in-language API, etc., are very similar to those of Nipype. I would like the authors to do a review and comparison of these tools, as well. Another tool or representation of potential interest could be the Common Workflow Language, which I only found brief mention of later on.

Page 3, Line 36, column 1

- Broken citation

Page 3, Line 48, column 1

- How does this provenance log compare to those obtained from Reprozip? The authors may wish to do a comparison of provenance standards in the first section, as well.

Figure 1

- What was the reason behind defining tasks in the way shown on lines 16 and 20? There are some standards, such as Boutiques (boutiques.github.io) and CWL which define task command-lines, including validating data typing, etc., that it seems could be of some use here to make sure that commands are being run meaningfully. For instance, these standards could perhaps enabling checking that all values in the DNA string are A, G, C, or T.
- I successfully re-executed this script after following the installation instructions found on the documentation page.

Page 6, Line 37-47, column 1

- Are you defining this provenance data with respect to any accepted standard, such as JSON-LD (the W3C-PROV compatible JSON format)? If not, how come, and what are the consequences of this custom definition of metadata?

Remove commas:

- Page 4, Line 48, column 2: after "used"
- Page 6, Line 25, column 2: after "workflow system"
- Page 7, Line 31, column 1: after "programming language"

Level of Interest

Please indicate how interesting you found the manuscript: Choose an item.

Quality of Written English

Please indicate the quality of language in the manuscript: Choose an item.

Declaration of Competing Interests

Please complete a declaration of competing interests, considering the following questions:

- Have you in the past five years received reimbursements, fees, funding, or salary from an organisation that may in any way gain or lose financially from the publication of this manuscript, either now or in the future?
- Do you hold any stocks or shares in an organisation that may in any way gain or lose financially from the publication of this manuscript, either now or in the future?
- Do you hold or are you currently applying for any patents relating to the content of the manuscript?
- Have you received reimbursements, fees, funding, or salary from an organization that holds or has applied for patents relating to the content of the manuscript?
- Do you have any other financial competing interests?
- Do you have any non-financial competing interests in relation to this paper?

If you can answer no to all of the above, write 'I declare that I have no competing interests' below. If your reply is yes to any, please give details below.

I declare that I have no competing interests

I agree to the open peer review policy of the journal. I understand that my name will be included on my report to the authors and, if the manuscript is accepted for publication, my named report including any attachments I upload will be posted on the website along with the authors' responses. I agree for my report to be made available under an Open Access Creative Commons CC-BY license (http://creativecommons.org/licenses/by/4.0/). I understand that any comments which I do not wish to be included in my named report can be included as confidential comments to the editors, which will not be published.

I agree to the open peer review policy of the journal

To further support our reviewers, we have joined with Publons, where you can gain additional credit to further highlight your hard work (see: https://publons.com/journal/530/gigascience). On publication of this paper, your review will be automatically added to Publons, you can then choose whether or not to claim your Publons credit. I understand this statement.

Yes Choose an item.
