# Supplementary material for: SciPipe: A workflow library for agile development of complex and dynamic bioinformatics pipelines
Source: Gigascience. 2019 Apr 26;8(5):giz044. doi: 10.1093/gigascience/giz044 (PMC6486472; doi:10.1093/gigascience/giz044)
Supplement: Reviewer_2_Report_Original_Submission -- Konstantinos Krampis, PhD — 11/2/2018 Reviewed [file giz044_reviewer_2_report_original_submission.pdf]

Reviewer Report

Title: SciPipe - A workflow library for agile development of complex and dynamic bioinformatics pipelines

Version: Original Submission **Date:** 11/2/2018

Reviewer name: Konstantinos Krampis, PhD

Reviewer Comments to Author:

Excellent work, building a workflow system implementing the data flow paradigm for bioinformatics with a fast language (GO) as well. Some minor concerns I have are the following:
- This is obviously targeted to bioinformatics developers, but assuming there is a community that adopts it, would there be a way for someone who can do basic command line to use
it ? I am assuming that is there is a community many workflows will be published and a non developer could run it with a single command by just pointing to his / her datasets ?
- What other dependencies does it need besides GO to be installed ?
- Can it do parallelism in data chunks similar to Nextflow ? Many bioinformatics files (think for example reads) can be processed in an embarrassingly parallel way, for example if they
are split and aligned to a reference genome.
- Finally, what about Docker containers ? For example Nextflow has build in the option to pull containers "on the fly" with the tools preconfigured from repositories such as Dockstore etc
which have hundreds of pre-made containers. This is especially useful in the case of complex bioinformatics pipelines which have 10-15 different tools. Of course a developer can build a single container with all the tools pre-configured and run SciPipe from within this container, but if container support is available natively within the SciPipe implementation, developers can simply point
to available (public or internally) containers with pre-configured tools, which will be started at runtime of the workflow in order to provide the algorithms which the workflow feeds the data
for processing during each different step.
Some corrections:
Line 33, right column better to use "Fig. 1" with bold letters.
Line 36, left column missing reference.
Line 46, right column, correct as "As can be seen on lines 17, 21 and 25, Fig.1", so that we know which figure we refer to.

Level of Interest

Please indicate how interesting you found the manuscript: Choose an item.

Quality of Written English

Please indicate the quality of language in the manuscript: Choose an item.

Declaration of Competing Interests

Please complete a declaration of competing interests, considering the following questions:

- Have you in the past five years received reimbursements, fees, funding, or salary from an organisation that may in any way gain or lose financially from the publication of this manuscript, either now or in the future?
- Do you hold any stocks or shares in an organisation that may in any way gain or lose financially from the publication of this manuscript, either now or in the future?
- Do you hold or are you currently applying for any patents relating to the content of the manuscript?
- Have you received reimbursements, fees, funding, or salary from an organization that holds or has applied for patents relating to the content of the manuscript?
- Do you have any other financial competing interests?
- Do you have any non-financial competing interests in relation to this paper?

If you can answer no to all of the above, write 'I declare that I have no competing interests' below. If your reply is yes to any, please give details below.

No competing interesting.

I agree to the open peer review policy of the journal. I understand that my name will be included on my report to the authors and, if the manuscript is accepted for publication, my named report including any attachments I upload will be posted on the website along with the authors' responses. I agree for my report to be made available under an Open Access Creative Commons CC-BY license (http://creativecommons.org/licenses/by/4.0/). I understand that any comments which I do not wish to be included in my named report can be included as confidential comments to the editors, which will not be published.

I agree to the open peer review policy of the journal

To further support our reviewers, we have joined with Publons, where you can gain additional credit to further highlight your hard work (see: https://publons.com/journal/530/gigascience). On publication of this paper, your review will be automatically added to Publons, you can then choose whether or not to claim your Publons credit. I understand this statement.

Yes Choose an item.
