# Supplementary material for: SciPipe: A workflow library for agile development of complex and dynamic bioinformatics pipelines
Source: Gigascience. 2019 Apr 26;8(5):giz044. doi: 10.1093/gigascience/giz044 (PMC6486472; doi:10.1093/gigascience/giz044)
Supplement: Reviewer_3_Report_Original_Submission -- Lars Ailo Bongo — 11/3/2018 Reviewed [file giz044_reviewer_3_report_original_submission.pdf]

Reviewer Report

Title: SciPipe - A workflow library for agile development of complex and dynamic bioinformatics pipelines

Version: Original Submission **Date:** 11/3/2018

Reviewer name: Lars Ailo Bongo

Reviewer Comments to Author:

##Comments to paper
This is a well written paper describing the, to my knowledge, first workflow manager implemented in Go. Although there are many alternative workflow managers, this work is motivated by the limitation of one of the state-of-the-art workflow mangers (Luigi) that the authors have previously used and even extended.
The paper describes the design of SciPipe, shows how it is used, and provides use cases. It does not provide any evaluation of SciPipe, nor does it describe system the use cases were run on. The latter should be included, since one of the motivations for SciPipe is the issues encountered with SciLuigi when run on more than 64 workers. The paper also does not describe how many users SciPipe has. Is it just used by the authors? I would also have liked a discussion about workflows, such as ADAM (https://github.com/bigdatagenomics/adam), that are implemented in Spark.
A minor issue: on page 3, line 36, there is a missing reference.
Comments to the source code and documentation
The SciPipe webpage is well designed, with documentation and example workflows. The GitHub repository has 833 commits, with the last commit on August 18th. It has 426 stars and 27 forks, which suggest that there is interest in the community. The install documentation are a bit hard to find in the webpage, especially for someone that has not read the paper. There does not seem to be a test suite for SciPipe.
I tested SciPipe on my laptop in Ubuntu on Windows. I have very limited knowledge of Go, so I just followed the examples on the webpage. They did work as described.
I first tested the RNA-seq case study. For it the documentation was less clear, and there were no instructions for how to do it. For example, how to specify the input dataset, which I later found was in the go code. The execution took a while, and it was hard to know if the program was working, or if it has crashed or waiting for input (especially since the first step downloads a 1.7GB file for which the size or progress is not shown). The case study failed, due to a missing library used by STAR. This is not a SciPipe issue, and it would not occur on a production system. SciPipe did however save the logs necessary to understand the issue.
Second, I tested the drug discovery workflow. It could not be compiled due to:
./utils.go:45: t.Round undefined (type time.Duration has no field or method Round)
Finally, I tested the genomics cancer workflow, which also failed due to a version issue in GenomeAnalysisTK.jar. Again, this is a third party installation error.

I did not do any more advanced testing of SciPipe, including using my own data, running it on more than one machine, nor stopping and restarting workflow execution.

Level of Interest

Please indicate how interesting you found the manuscript: Choose an item.

Quality of Written English

Please indicate the quality of language in the manuscript: Choose an item.

Declaration of Competing Interests

Please complete a declaration of competing interests, considering the following questions:

- Have you in the past five years received reimbursements, fees, funding, or salary from an organisation that may in any way gain or lose financially from the publication of this manuscript, either now or in the future?
- Do you hold any stocks or shares in an organisation that may in any way gain or lose financially from the publication of this manuscript, either now or in the future?
- Do you hold or are you currently applying for any patents relating to the content of the manuscript?
- Have you received reimbursements, fees, funding, or salary from an organization that holds or has applied for patents relating to the content of the manuscript?
- Do you have any other financial competing interests?
- Do you have any non-financial competing interests in relation to this paper?

If you can answer no to all of the above, write 'I declare that I have no competing interests' below. If your reply is yes to any, please give details below.

I declare that I have no competing interests

I agree to the open peer review policy of the journal. I understand that my name will be included on my report to the authors and, if the manuscript is accepted for publication, my named report including any attachments I upload will be posted on the website along with the authors' responses. I agree for my report to be made available under an Open Access Creative Commons CC-BY license (http://creativecommons.org/licenses/by/4.0/). I understand that any comments which I do not wish to be included in my named report can be included as confidential comments to the editors, which will not be published.

I agree to the open peer review policy of the journal

To further support our reviewers, we have joined with Publons, where you can gain additional credit to further highlight your hard work (see: https://publons.com/journal/530/gigascience). On publication of this paper, your review will be automatically added to Publons, you can then choose whether or not to claim your Publons credit. I understand this statement.

Yes Choose an item.
